# Malignant mixed mullerian tumors: a SEER database review of rurality and treatment modalities on disease outcome

**DOI:** 10.3389/fonc.2024.1296496

**Published:** 2024-02-08

**Authors:** Neusha Zadeh, Arjun Bhatt, Vaishnavi Sripiparu, Melisa Pasli, George Edwards, Michael C. Larkins, M. Sean Peach

**Affiliations:** ^1^ Jerry M. Wallace School of Osteopathic Medicine, Campbell University, Buies Creek, NC, United States; ^2^ Brody School of Medicine, East Carolina University, Greenville, NC, United States; ^3^ Department of Radiation Oncology, Brody School of Medicine, East Carolina University, Greenville, NC, United States

**Keywords:** SEER analysis, mixed mullerian tumors, uterine carcinosarcomas, rural cancer, gynecologic cancer

## Abstract

**Introduction:**

Malignant Mixed Mullerian Tumors (MMMT) are rare and poorly understood sarcomas with limited research on risk factors, pathogenesis, and optimal treatments. This study aimed to address this knowledge gap and explore the impact of community size, patient characteristics, disease characteristics, and treatment modalities on MMMT outcomes.

**Methods:**

Using the Surveillance, Epidemiology, and End Results database (SEER), the largest SEER cohort to date of 3,352 MMMT patients was analyzed for demographic factors, treatment modalities, and histologic characteristics. Data was processed, including the removal of incomplete entries, and analyzed in Python 3.1 using packages *scikit-learn, lifelines*, and *torch*; log-rank analysis and Cox proportional hazards models were used to evaluate a number of demographic characteristics and disease characteristics for significance in regard to survival.

**Results:**

Our study found adjuvant radiotherapy and chemotherapy significantly improved survival, with modest benefits from neoadjuvant chemotherapy. Our findings also suggest age at diagnosis, disease grade, and suburban versus rural geographic locations may play key roles in patient prognosis. On multivariable analysis both disease Grade and surgical treatment were significant factors.

**Discussion:**

MMMTs remain challenging, but appropriate treatment appears to enhance survival. The present findings suggest opportunities for improved outcomes and treatment strategies for patients with MMMTs.

## 1 Introduction

Malignant Mixed Mullerian Tumors (MMMTs), also known as uterine carcinosarcomas, are rare and aggressive tumors that arise in the genital tract of postmenopausal women. They comprise 5% of all uterine neoplasms and 16.4% of all uterine cancer-related deaths (1, 2). Despite increased research interest in the pathologic mechanisms of MMMTs, risk factors, late diagnosis, and variable access to treatment have further contributed to a poor prognosis. Further prognostic factors include increasing age, lymph node metastasis, suboptimal surgical cytoreduction, the presence of heterologous features on histopathology, and heightened expression of VEGF, tumor protein p53, and p53 coupled with Wilms tumor 1 (WT1; 3). Current literature suggests that the five-year overall survival (OS) rate is less than 35%, which is a stark contrast to the 76% survival rate of endometrial stromal sarcomas (4, 5).

The biphasic histology of MMMT’s entails both malignant epithelial and mesenchymal components commonly found in the uterus but sometimes arising in the ovaries, fallopian tubes, or vagina. The diagnosis of MMMTs is challenging due to its varied clinical presentation, which may include, but is not limited to, abnormal vaginal bleeding, bloody or watery discharge, abdominal or pelvic pain, and palpable pelvic masses (6).

The absence of any highly sensitive or specific clinical signs of this malignancy may inform why it is typically discovered relatively late on initial presentation: approximately one-third of patients possess clinical manifestations of positive regional lymph node metastasis, while the incidence of visceral metastasis at presentation is roughly 10% (7). Therapeutic options decrease with advanced disease; treatment in the presence of distant metastases is generally palliative (8, 9). Surgical intervention is indicated in masses > 6 cm or in symptomatic masses. Controversy exists regarding the notion that the epithelial component of the tumor drives metastasis as there is evidence for independent and separate metastatic potential of the two histologic components, and histological analyses of metastases have demonstrated mixed results (6, 10). The uncertainty surrounding the mechanism of metastases and the high mortality associated with disease spread underscore the importance of screening and diagnostic tools in identifying malignancy at earlier stages.

Previous systematic reviews have identified older age, Black race, obesity, long-term tamoxifen use, and prior pelvic radiation as risk factors for the development of MMMTs (9, 11–13). Previous SEER analyses have reviewed MMMT risk factors, such as medical predispositions, race, and socioeconomic variables. These studies compared the incidence, prognosis, and survival associated with different treatment modalities of uterine carcinosarcomas with carcinosarcomas of the cervix and ovaries and compared adjuvant chemoradiation to intraoperative lymphadenectomy for optimal disease control. However, none of these analyses have investigated variables such as geography, the influence of diagnostic methods of varying accuracy, treatment and survival in the non-surgical candidate, nor the role of neoadjuvant therapy. Furthermore, the largest SEER analysis of treatment trends prior to the present examination consisted of 1,541 patients (11). Our aim was to provide insight into the prognosis and OS of patients stratified by age, community residency, and available therapeutic options across chemotherapeutic, radiotherapeutic, and surgical modalities. We further offer brief analyses into treatment modalities that prolong survival in non-surgical candidates, current treatment trends in regard to adjuvant versus neoadjuvant chemotherapy and radiation, and provide an update to previously examined demographic, tumor, and treatment variables based on a twofold larger sample size on the most recent SEER data to date.

## 2 Materials and methods

This is a retrospective cohort study utilizing the Surveillance, Epidemiology and End Results (SEER) database sponsored by the National Cancer Institute (NCI; 14). This registry provides deidentified, detailed disease course data spanning approximately 28% of the US population. The registry was queried as a “Case Listing Session” for all 667 patient cases where variable “Histologic Type” was of “Malignant Mixed Mullerian Tumors”. Patients were diagnosed with MMMT between 2000 and 2018. Exclusion criteria included patients with multiple primary fields, patients with an unknown or unclear diagnosis, and patients under 15 years old at the time of diagnosis.

Variables selected for review included patient demographics such as race, age, and geography, as well as tumor characteristics, including grade, tumor type, and histopathologic characteristics. Treatment modalities, such as surgical resection, radiation therapy, systemic therapy, and other adjunctive therapies were considered in conjunction with Kaplan Meier survival outcomes for various combinations and orders of the aforementioned treatment types. Outcomes based on tumor grade and histological characteristics were also analyzed. Data was processed, including the removal of incomplete entries, and analyzed in Python (Version 3.1; Scotts Valley, CA: CreateSpace) using packages *scikit-learn*, *lifelines*, and *torch*. Statistical analysis was conducted using the aforementioned Python packages and SPSS (Version 27.0; Armonk, NY: IBM Corp.), with a p-value of < 0.05 deemed statistically significant. Log rank analysis and Cox proportional hazards models were used to evaluate a number of demographic characteristics and disease characteristics for significance in regard to survival.

Diagnostic confirmation was interpreted in line with SEER coding standards. Briefly, tumors were coded as histologically diagnosed if microscopic diagnosis was based on fine needle aspirate, biopsy, surgery, autopsy, or dilation and curettage (D&C). Tumors were considered cytologically diagnosed if the patient was diagnosed via peritoneal fluid or cervical or vaginal smears. Histological diagnoses were also coded as higher priority compared to cytological diagnoses.

## 3 Results

### 3.1 Demographics

**Figure 1:**
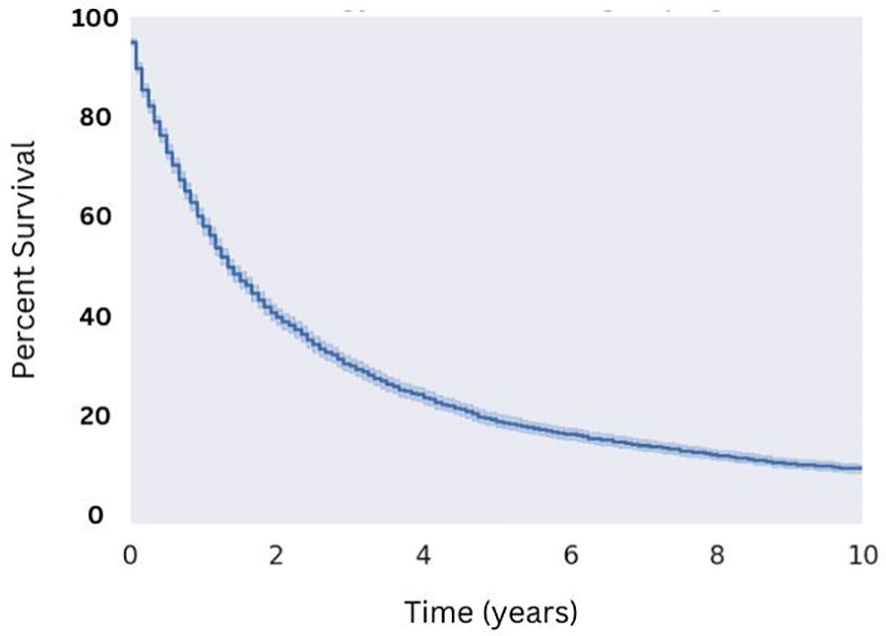
Overall Survival for Patients with MMMT. N=3,352 patients were extracted from the SEER database from the 2000 to 2018 reporting years with the diagnosis of MMMT, yielding a median OS of 16 months.

**Figure 2:**
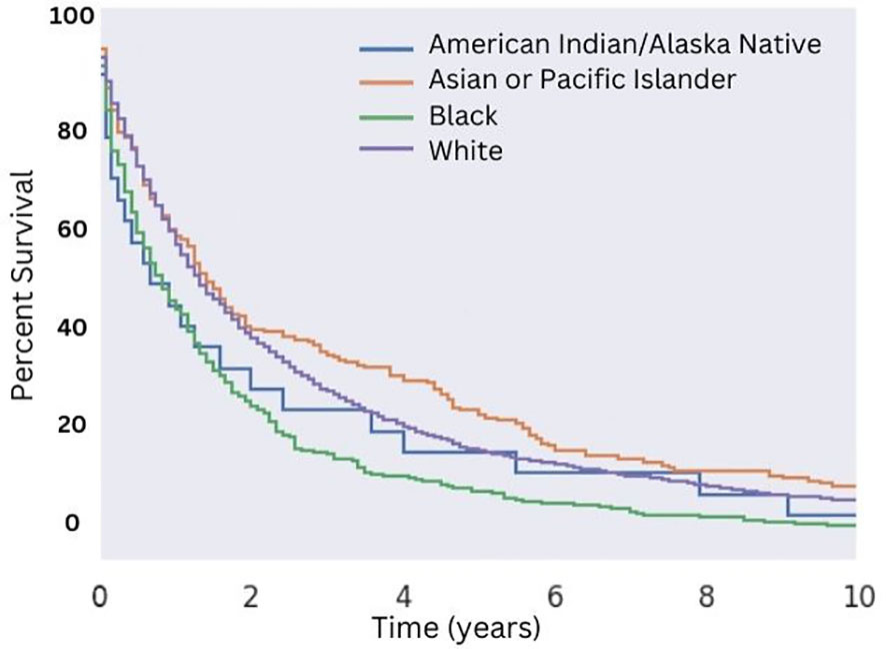
Overall Survival for Patients with MMMT by Race. OS of patients diagnosed with mixed mullerian malignant tumors in the 2000-2018 SEER database were stratified by race. There was no significant difference in median OS (p > 0.05) across all races. An increased median OS of 16 months versus 13 months for White patients and non-White patients, respectively, was seen (p = 0.03).

**Figure 3:**
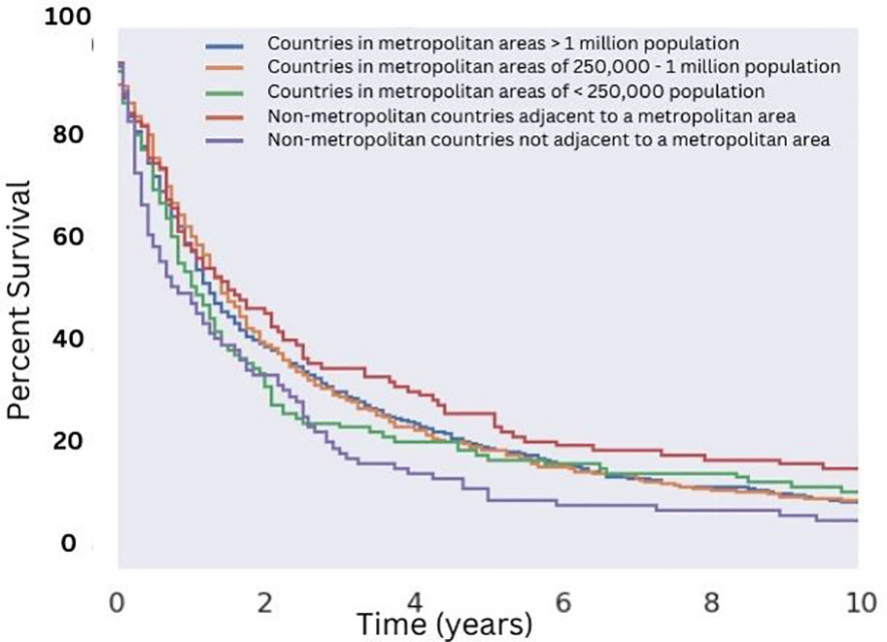
Overall Survival for Patients with MMMT by Urban-Rurality Status. OS of patients diagnosed with mixed mullerian malignant tumors in the 2000-2018 SEER database were stratified by Urban-Rurality county classification. Patients from non-metropolitan counties not adjacent to a metropolitan area had the lowest median survivorship (12 months). Patients from nonmetropolitan counties adjacent to a metropolitan area had the greatest median survivorship (20 months), which was significant compared to the aforementioned group (p < 0.05).

Utilizing selection criteria as detailed above, we obtained 3,352 cases of MMMTs. The median age at diagnosis was 64.3 years, ranging from patients between 15 and 20 years old to those older than 85. MMMTs were most prevalent in Caucasians, representing 84% of our cohort, followed by African Americans at 8.7%, Asian and Pacific Islanders at 6%, and American Indians or Alaska Natives at 0.7%. Race and other demographic information are summarized in **Table 1**. Patients with MMMTs had a median overall survival of 16 months with a range between zero months and 19 years (**Figure 1**). There were no differences in OS when comparing patients stratified by race (**Figure 2**). Exploring results categorized by rural/urban categorization provided deeper insight into the discrepancies between rural and urban communities. Kaplan Meier analysis demonstrated that nonmetropolitan counties not adjacent to a metropolitan area had the lowest overall survivorship rates with a median survivorship of 12 months. Conversely, nonmetropolitan counties adjacent to a metropolitan area had significantly greater survival with a median of 19.5 months (p < 0.05) as illustrated in **Figure 3**.

**Table 1:** Demographic Characteristics & Corresponding Survival of MMMT.

Variable	N	Median Survival in months (min, max)
Race		
White	2822	16 (0, 227)
Black	292	10 (0, 210)
Asian or Pacific Islander	200	18 (0, 223)
American Indian / Alaska Native	24	9.5 (0, 166)
Urban-Rural Status		
Metropolitan Counties: > 1 million population	676	16 (0, 354)
Metropolitan Counties: 250,000 to 1 million population	422	18 (0, 279)
Metropolitan Counties: < 250,000 population	110	13.5 (0, 351)
Nonmetropolitan Counties adjacent to Metropolitan area	110	19.5 (0, 324)
Nonmetropolitan Counties not adjacent to Metropolitan area	97	12 (0, 336)
Age		
25 - 29 year olds	10	63 (2, 151)
30 - 34 year olds	10	86 (28, 220)
35 - 39 year olds	28	22 (1, 221)
40 - 44 year olds	85	29 (1, 226)
45 - 49 year olds	152	22 (0, 224)
50 - 54 year olds	283	21 (0, 227)
55 - 59 year olds	385	20 (0, 222)
60 - 64 year olds	458	18.5 (0, 210)
65 - 69 year olds	531	17 (0, 226)
70 - 74 year olds	495	15 (0, 219)
75 - 79 year olds	423	13 (0, 210)
80 - 84 year olds	312	10 (0, 194)
85 + year olds	176	5.5 (0, 154)
Tumor Grade		
Well Differentiated; Grade I	29	130 (0, 221)
Moderately Differentiated; Grade II	51	43 (1, 219)
Poorly Differentiated; Grade III	1045	19 (0, 226)
Undifferentiated; Grade IV	695	18 (0, 226)
Diagnostic Confirmation		
Positive Histology	3245	16 (0, 227)
Positive Exfoliative Cytology, No positive Histology	95	8 (0, 193)
Cancer-Directed Surgery		
Surgery performed	2942	18 (0, 227)
Not recommended	324	5 (0, 224)
Not recommended; contraindicated due to other condition	32	2 (0, 20)
Recommended but not performed; unknown reason	20	17.5 (0, 98)
Recommended but not performed; refused	12	5.5 (0, 27)
Recommended; unknown if performed	14	19 (2, 61)
Radiation		
Standard Fractionation External Beam Radiation Therapy	98	21.5 (1, 222)
None/Unknown	3219	16 (0, 227)
Chemotherapy		
Yes	2413	20 (0, 227)
None/Unknown	937	5 (0, 226)
Neoadjuvant vs. Adjuvant Therapy		
Radiation after surgery	93	22 (1, 222)
Systemic therapy before surgery	122	17 (0, 131)
Systemic therapy after surgery	1314	21 (0, 153)
Systemic therapy both before and after surgery	164	20.5 (0, 96)
Surgery both before and after systemic therapy	6	25 (7, 34)
No radiation and/or cancer-directed surgery	3250	16 (0, 227)
No systemic therapy and/or surgical procedures	749	5 (0, 155)

Table depicting various demographic characteristics for patients diagnoses with malignant mixed Mullerian tumors based on analysis of the Surveillance, Epidemiology, and End Results (SEER) Program.

### 3.2 Tumor characteristics

**Figure 4:**
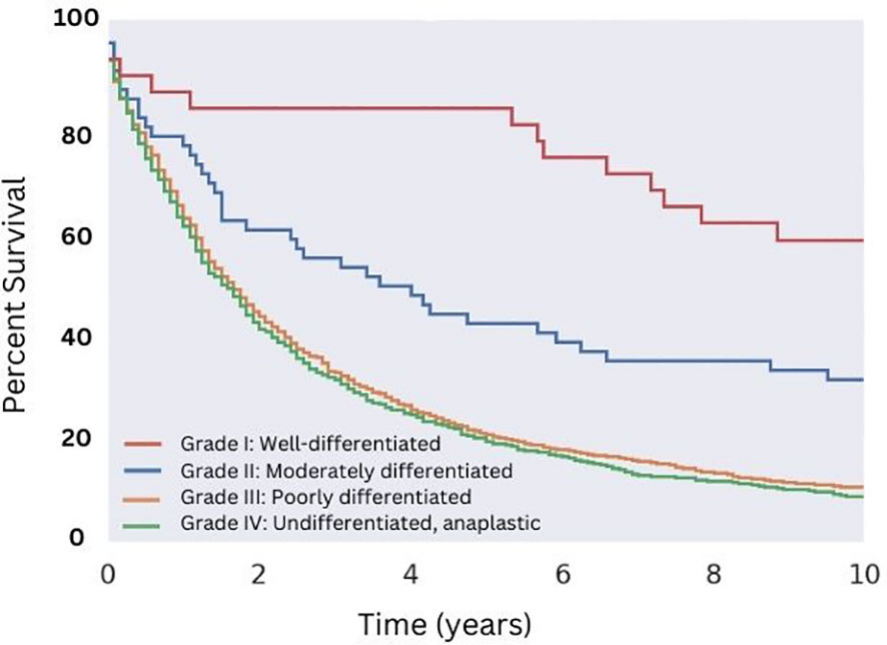
Overall Survival for Patients with MMMT by Histologic grade. OS of patients diagnosed with mixed mullerian malignant tumors in the 2000-2018 SEER database were stratified by histologic grade. Well-differentiated disease showed the highest median OS of 130 months (p < 0.05), followed by moderately differentiated disease (median OS of 43 months, p < 0.05). Both poorly differentiated and undifferentiated disease showed no difference in median OS of approximately 19 and 18 months, respectively (p = 0.13).

Regarding characteristics of the malignancy itself, we found a large majority of cases (86%) exhibited high tumor grade, and that grade and differentiation significantly impacted survivorship. Well-differentiated (Grade I) and moderately differentiated (Grade II) carcinosarcomas demonstrated greater OS relative to poorly differentiated (Grade III) and undifferentiated (Grade IV) carcinosarcomas (**Figure 4**). Patients with Grade I disease saw a significantly improved median OS among all groups (10.8 years), compared to a median OS of 43 months for patients with Grade II disease, 19 months for patients with Grade III disease, and 18 months for patients with Grade IV disease.

### 3.3 Diagnostic methods

Patients who received a histological diagnosis demonstrated a significantly greater OS compared to those who were diagnosed via cytology (p < 0.05). Only 20% of patients with a cytological diagnosis received surgery, compared to 90% of patients with a histological diagnosis. 8% of patients with a histological diagnosis did not undergo surgery, while 62% of patients with a cytological diagnosis did not undergo surgery. There was no statistically significant difference in survival following surgery between patients who received a histological diagnosis and patients who did not. Cytological specimens were obtained via endometrial sampling from patients and submitted for diagnostic testing.

### 3.4 Treatment analysis

**Figure 5:**
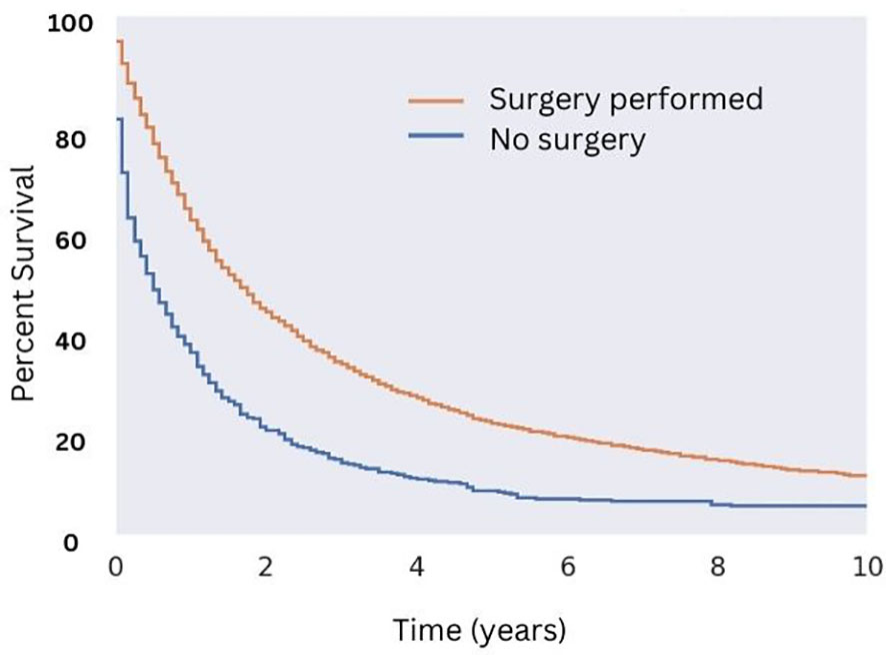
Overall Survival for Patients with MMMT by Surgical Status. OS of patients diagnosed with mixed mullerian malignant tumors in the 2000-2018 SEER database were stratified by presence or lack of surgical management. Patients who underwent surgery saw an increase in median OS (18 months versus five months, p < 0.05) and 10-year OS (10% versus 1% for those who did not undergo surgery; p < 0.05).

**Figure 6:**
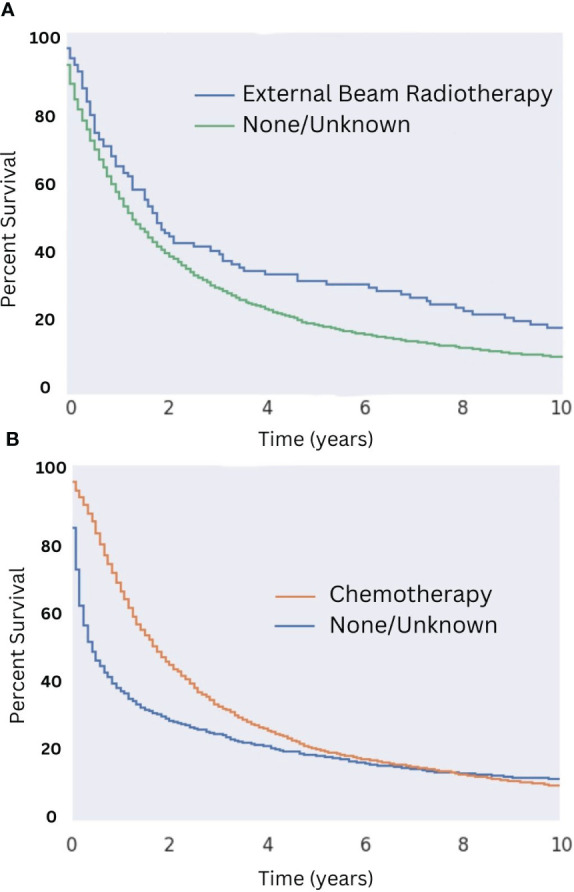
Overall Survival for Patients with MMMT by Radiotherapy and Chemotherapy Status in those who underwent Surgery. OS of patients diagnosed with mixed mullerian malignant in the 2000-2018 SEER database was stratified by presence or lack of radiotherapy **(A)** and chemotherapy **(B)** in those who underwent surgery. Patients who received external beam radiation therapy saw an increased 10-month OS of 17% compared to those who did not (10-month OS of about 9%; p < 0.05). Median OS was 22 months for patients who received radiotherapy, compared to 17 months for those who did not. No difference in 10-month OS was seen between patients who did or did not receive chemotherapy. Median OS in patients who received both surgery and chemotherapy was 21 months, compared to seven months for those who received surgery but did not receive chemotherapy.

Patients who underwent surgical resection of well-differentiated tumors had significantly greater median OS compared with those that did not (18 months for those that received surgery compared to five months for those that did not; **Figure 5**). While there were a variety of radiotherapeutic modalities used to treat MMMTs, including External Beam Radiation Therapy (EBRT), brachytherapy, and the combination of the two, EBRT was by far the most popular (approximately 75% of patients who received radiation therapy). Patients who received EBRT lived significantly longer than those who did not (**Figure 6A**, p < 0.05 in regards to median OS). Systemic chemotherapy also prolonged survival, however, any survival benefit of chemotherapy did not appear to persist beyond 5 months (**Figure 6B**).

#### 3.4.1 Adjuvant and neoadjuvant analysis

**Figure 7:**
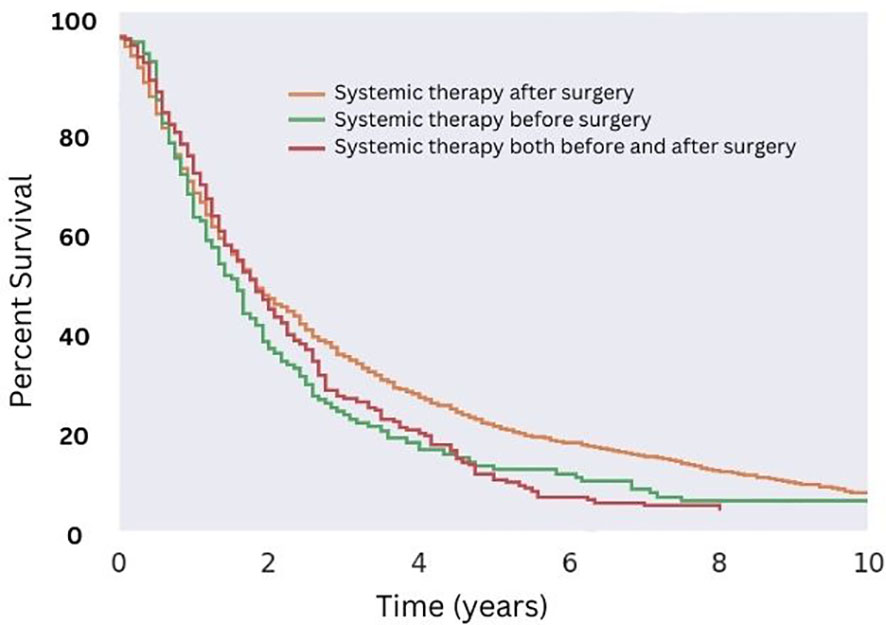
Overall Survival for Patients with MMMT by Neoadjuvant vs Adjuvant Chemotherapy Status. OS of patients diagnosed with mixed mullerian malignant tumors in the 2000-2018 SEER database were stratified by timing of adjuvant chemotherapy vs surgery. Patients received adjuvant, neoadjuvant, or both adjuvant and neoadjuvant chemotherapy. Adjuvant therapy showed improved OS at five years (p < 0.05) though this trend did not persist at the 10-year mark.

Radiation therapy was used as both adjuvant and neoadjuvant therapy, as well as delivered intraoperatively for the management of MMMTs. Due to low sample size of patients treated with intraoperative and neoadjuvant radiation (four patients total) this limited any analysis to determine if timing and or modality had a significant effect. Similarly, systemic chemotherapy was also given in both the adjuvant and neoadjuvant setting. Adjuvant chemotherapy appeared to be most effective in prolonging OS (p < 0.05), with an increase of about 8% at the five-year mark compared to those who received neoadjuvant therapy (p < 0.05; **Figure 7**). This trend of increased survival did not persist approaching the 10-year mark. Patients receiving both adjuvant and neoadjuvant therapy fared the worst, with all patients dying within eight years of diagnosis.

### 3.5 Non-surgical candidate analysis

**Figure 8:**
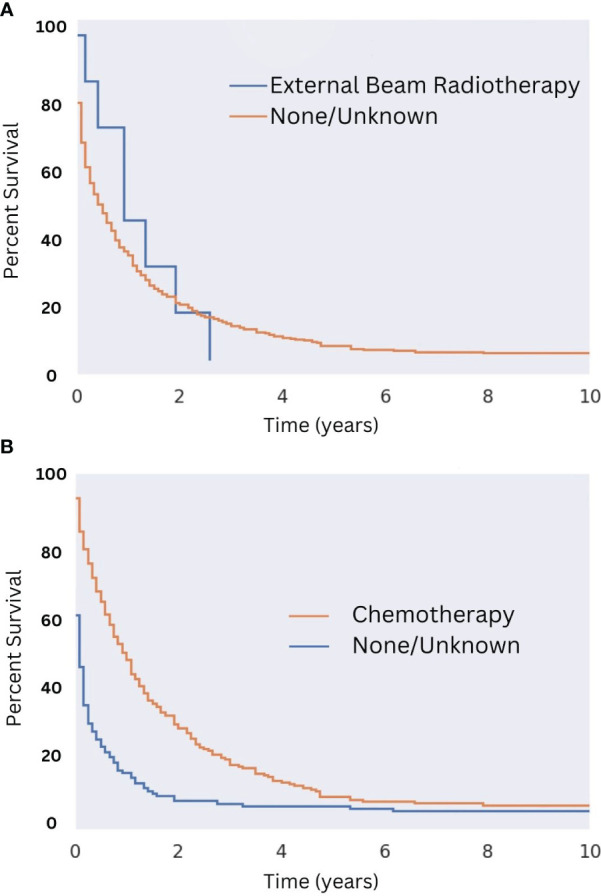
Overall Survival for Patients with MMMT by Radiotherapy and Chemotherapy Status in those not undergoing Surgery. OS of patients diagnosed with mixed mullerian malignant tumors in the 2000-2018 SEER database were stratified presence or lack of radiotherapy **(A)** and chemotherapy **(B)** in those who are not surgical candidates. Radiotherapy was not seen to improve OS (n = 8; p = 0.44). Chemotherapy improved OS slightly (2% improvement; p < 0.05). This survival benefit was most notable in the first five years following diagnosis with a median survival being one month for patients not treated with chemotherapy and 12 months for those who were (p < 0.05).

Among patients who were not surgical candidates, the available data suggest that chemotherapy (median survival of 12 months) but not radiation (median survival of 11 months) provided a survival benefit (see **Figures 8A, B**, respectively). The benefit of radiation among patients who did not receive surgery may be obscured by the low sample size, as only eight patients who did not undergo surgery received radiation. The survival benefit afforded by chemotherapy in this population was most notable in the first five years after diagnosis with median survival being one month for patients not treated with chemotherapy and 12 months for those that were (**Figure 8B**; p = 0.44). Of patients who did not receive surgery, 40% did not receive either chemotherapy or radiation. The median survival for this subset of patients was five months.

### 3.6 Overall trends and multivariable analysis

**Figure 9:**
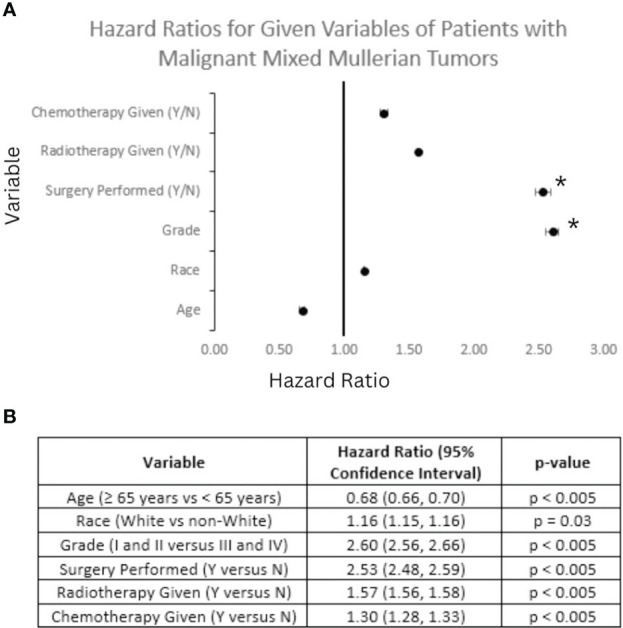
Forest Plot of Selected Variables and Multivariable Analysis. Hazard ratio (HR) calculations for selected variables based on overall survival (OS) of patients diagnosed with mixed mullerian malignant tumors between 2000 and 2018 based on analysis of the SEER database. **(A)** A Forest plot of selected variables highlights that on multivariable analysis surgical status and Grade I or II disease (denoted with a *) maintained significance in regards to OS. **(B)** Table depicting the breakdown of HR and 95% confidence intervals for each variable in the Forest plot above. All HR calculations met significance criteria.

Univariant analysis was performed in regards to overall survival. Hazard ratios (HR) for selected variables in this analysis are displayed in **Figure 9**. It can be seen that patients older than 65 years of age (HR: 0.68; p < 0.05) and non-White patients (HR: 1.16; p < 0.05) saw decreased OS compared to patients diagnosed with MMMT at less than 65 years of age and White patients, respectively. Patients with Grade I or II disease saw increased OS compared with those diagnosed with Grade III or IV disease (HR: 2.60; p < 0.05). Patients treated with chemotherapy (HR: 3.00; p < 0.05) and radiotherapy (HR: 1.57; p < 0.05) both saw increased OS, compared to those that did not receive these treatments. Patients treated with surgery saw the greatest increase in OS for all treatment modalities (HR: 2.53; p < 0.05) compared to those that did not. Multivariable analysis controlling for treatment modality, disease grade, patient age, and patient race showed significance with only disease grade and surgical treatment of MMMT.

## 4 Discussion

### 4.1 Literature review

As of today, histological examination of tissue samples remains the gold-standard for diagnosis. In the literature, 75% of MMMTs are misdiagnosed as adenocarcinoma preoperatively given MMMTs biphasic nature and correct surgical staging often requiring large tissue samples (15). A negative endometrial biopsy does not rule out diagnosis as it does not obtain sufficient tissue (16). The use of early tumor imaging is of benefit for suspected patients with uterine cavities not readily accessible for biopsy but can be nonspecific in differentiating one tumor from another (17). Exfoliative cytology may provide confirmation if a biopsy is not available or appropriate. However, this method has variable and limited sensitivity, with 60% of tests correctly detecting some form of malignancy and only 8.6% of cytology specimens correctly detecting MMMTs specifically, as opposed to mistaking MMMTs for adenocarcinoma (17, 18). Therefore, histopathological confirmation is important even in those that are not surgical candidates as correct diagnosis has significant implication on prognosis and management.

Now considered an atypical histology by most treatment algorithms, MMMTs are overall treated similarly to high-grade endometrial adenocarcinoma. Endometrial adenocarcinoma is a relatively well-understood disease, and treatment guidelines fractionate patients very finely based on disease stage and grade. Inherent to their biphasic histology, MMMTs are more complex than endometrial adenocarcinoma, with treatment guidelines more nuanced than those of endometrial adenocarcinomas— indeed, differences in the pathophysiology of disease spread between high grade endometrial cancers and MMMTs have been recognized for nearly 20 years (19). MMMTs exhibit resistant behavior against conventional chemotherapies and have a high rate of recurrence— projected to be 37% and 46% at stages I and II respectively, with further increase as the disease progresses. This has been attributed to the disease’s histological features, including high-grade epithelial and sarcomatous qualities (6, 20). While a complete surgical resection remains the primary approach, the ongoing debate regarding the optimal treatment regimen stems from suggestions to improve treatment paradigms by offering different modalities based on the stage at presentation and more precisely evaluating surgical candidacy in the context of patient risk factors (21, 22). Adjuvant treatment recommendations including chemotherapy and radiation, also remain unclear, as does disease prognosis in the setting of adjuvant treatments (23).

Due to their rarity, MMMTs have remained understudied in terms of identifying contributing risk factors and understanding their effects on pathogenesis. In an effort to address this knowledge gap, we conducted the largest study to date specifically focusing on MMMTs, with a particular emphasis on community size, the utilization of adjuvant/neoadjuvant treatments, and the care of nonsurgical candidates. We identify patients from suburban areas as having the best disease outcomes, and patients from rural areas as having the worst. We also address the paucity of data in the literature regarding the role of adjuvant and neoadjuvant therapies, finding significant improvements in survival associated with both adjuvant radiotherapy and chemotherapy, and a more modest improvement associated with neoadjuvant chemotherapy (9, 13). While there is existing literature and well-established guidelines concerning the utilization of combined therapies for endometrioid adenocarcinomas, our study aims to provide findings that encourage further exploration of similar treatment regimens for MMMTs (24). This knowledge has the potential to improve patient outcomes, guide treatment decisions, and pave the way for the development of more effective therapeutic strategies for this rare disease.

### 4.2 Rurality

Current literature focusing on cancer incidences and patient rurality statuses collapse the multilevel variability of rurality status into two broad categories (metropolitan vs. non-metropolitan) due to small numbers in some strata limiting the increasing number of subcategories (25). However, this measure of rurality may mask differences amongst subgroups within each group and could shroud potential variables differentiating various metropolitan and nonmetropolitan areas. Our study further explores the variable nuances of metropolitan and nonmetropolitan populations through the differentiation of various population sizes in metropolitan areas and the general proximity status to larger metropolitan counties for nonmetropolitan areas. Results show that nonmetropolitan counties adjacent to metropolitan counties did not show a significant difference in overall survival when compared to metropolitan counties. However, a statistically significant difference of overall survival was found between nonmetropolitan counties not adjacent to metropolitan counties and large metropolitan counties (p < 0.05).

These results could be the outcome of limited resource availability for isolated nonmetropolitan counties not adjacent to metropolitan counties when compared to nonmetropolitan areas with large metropolitan infrastructure nearby. Previous studies have supported that those residing in isolated rural communities have higher barriers to pivotal treatment modalities. These barriers, such as increased travel distance, decreased professional access, and decreased health literacy, often increase in severity and prevalence further rural communities are from larger metropolitan areas (26). Additionally, it has been found that limitations of access to modern cancer treatment modalities and screening mechanisms in isolated rural communities can lead to overall higher cancer staging at diagnosis and poorer average prognosis (27–29). In our study, this trend of rural patients presenting with later stage disease was noted but not significant, likely due to the low number of MMMT patients who also lived in rural areas. It is probable that the many barriers of isolated patient rurality status contribute to the worse survival outcomes of patients from rural counties further away from higher population metropolitan counties.

### 4.3 Diagnostic methods

Cytology stands as a valuable initial screening tool owing to its non-invasive nature, rapid and ready accessibility and cost-effectiveness. However, cytology often fails to offer conclusive results or shows a limited picture of the condition at hand. This presents a challenge in its utilization compared to histology, notably due to the variable pathological presentation of MMMTs. Our research also demonstrated a positive histological diagnosis was associated with improved survival relative to patients diagnosed with cytology. Further investigation revealed patients with positive histology were significantly more likely to be offered surgery than patients with positive cytology. As a result of SEER coding convention, and in the clinical context of uterine and endometrial carcinomas, a histological diagnosis may refer to either a biopsy/D&C, or a histological examination of a surgical specimen. Furthermore, a patient who received both a cytological and surgical pathology diagnosis would be coded only as having received a histological diagnosis. Given this ambiguity, it is difficult to construct a causal understanding of the relationship between surgical candidacy and diagnostic method: it may be that all patients initially received a cytological diagnosis, and only the subset of those patients that were surgical candidates received a histological diagnosis in the form of a surgical pathology examination, thus making cytologic diagnosis a proxy of surgical candidacy. On the other hand, patients with only cytologic pathological analysis were likely not surgical candidates due to advance disease stage or comorbidities.

### 4.4 Treatment

Surgical resection, which by standard is a total abdominal hysterectomy with bilateral salpingo-oophorectomy (TAH-BSO) with or without nodal evaluation, is favored as the initial treatment approach and is generally considered a sufficient therapy option for patients diagnosed with an early stage MMMT (21, 22). Prior studies performed exploring the reasons behind cancer patient refusal of recommended cancer-directed treatment have shown that patients’ socioeconomic conditions, reflected by marital status and medical insurance plan, can play a significant role in the decision-making process (30, 31). It is possible that patients are refusing therapies and surgeries due to the cost and resulting financial burdens these procedures can create. Other articles have also explored the effect of rurality on patient refusal. Particularly, patient residence in a rural county has displayed an association with increased health care avoidance behaviors (32), a specific association that was not able to be investigated in the present study

Chemotherapy was the most frequently utilized treatment modality within the subset of patients in our study who were not surgical candidates. Among non-surgical candidates, often attributable to metastatic disease, extensive pelvic nodal involvement, or presence of medical comorbidity, our data suggests chemotherapy may still offer a survival benefit of approximately six additional months. Examination of the prognosis of patients with recurrent MMMT revealed salvage therapy utilizing radiotherapy, chemotherapy, and chemoradiotherapy (CRT) all contributed to improved survival after recurrence (SAR) and cancer-specific survival (CSS). Notably, while surgical intervention improved CSS, it did not significantly improve SAR (33). In line with our own study findings, the literature supports the potential of chemotherapy to extend life in this patient population (33). Therefore, non-surgical modalities may be of benefit for patients with MMMT in recurrent circumstances.

Lastly, targeted radiation may have a role in the palliative setting based on tumor growth patterns and/or metastasis. The conclusions drawn from studies on MMMT indicate that the palliative care approach responds to individual patient needs rather than constituting an integral aspect of routine diagnostic care. Patients receiving any modality of treatment, be it for curative or palliative intent, with or without surgery, have been shown exhibit better overall survival rates (34). Palliative interventions to alleviate symptoms included radiotherapy and chemotherapy. Referrals for palliative care were frequent among patients experiencing recurrent or progressive disease rather than those demonstrating resistance to adjuvant therapy (35). This approach aimed to manage symptoms, facilitate hospice care, and assist in end-of-life care decision-making. As such, numerous non-surgical interventions remain available for patients with aggressive malignancies, but further research is warranted to explain these findings.

Advancements to treatment options bring hope in increasing OS relative to patients’ age of diagnosis, initial MMMT staging, and post-surgical outcomes. Our findings suggest adjuvant radiation therapy provided an independent survival benefit, both overall and in the subpopulation of patients who received chemotherapy (p < 0.05). Our results also showed a survival benefit associated with chemotherapy among the subset of patients who were not surgical candidates. This suggests multimodal therapy may offer benefit for patients with MMMTs, and based on individual patient tolerability, perhaps even for patients with inoperable tumors.

The use of adjuvant chemotherapy and radiation therapy has both increased over time and improved the OS of patients with MMMTs (7). A study of older MMMT patients did not find sufficient data to suggest restricting adjuvant radiation (p = 0.28) or chemotherapy (p = 0.61) as further treatment options, even in late-stage disease (36, 37). Similarly, the present examination found adjuvant chemotherapy and radiation to both individually offer a survival benefit. Neoadjuvant chemotherapy was found to offer a small survival benefit, but given the larger benefit of adjuvant therapy, and the importance of not delaying surgical treatment, clinicians should exercise caution in therapeutic planning for the neoadjuvant setting. These results are in line with a study in Stage IV MMMT patients, which found no significant improvement in OS when comparing the use of neoadjuvant therapy followed by resection in initially inoperable patients to candidates who underwent primary surgery (38).

### 4.5 Limitations and concluding remarks

Our study has several limitations that should be acknowledged. First, due to its retrospective nature, there are inherent limitations associated with analyzing previously collected data. There were instances of missing or unknown data, particularly regarding the grade and stage of tumors and cause of death, which, if there were a pattern to the missing values, could affect the accuracy of our analyses. Another weakness is that the SEER database is observational and cannot establish causality between variables, nor can it assess treatment details such as radiation doses, specific chemotherapeutic regimens, or medical comorbidity. For example, in non-operative patients there was no benefit to OS with radiotherapy, however, there is no way in the SEER database to distinguish if definitive radiotherapy was attempted vs palliation to a metastasis, diluting any potential effect that aggressive radiotherapy may have in non-operative patients. Further, patient follow-up data is limited and data regarding post-treatment complications, functional impairments, and patient-reported outcomes are not available through this database and may be a source of potential future investigations.

The present work indicates surgery and adjuvant chemotherapy and/or radiotherapy as important determinants for improved OS for MMMT. Neoadjuvant therapy remains relatively uncommon and offers only a modest benefit relative to the absence thereof and portends significantly worse outcomes compared to adjuvant therapies. For those who are not surgical candidates, the present analysis demonstrates that radiotherapy and/or chemotherapy improve OS in those who are not surgical candidates. Current risk factors for poor treatment outcomes have expanded in the present work to include rural patients adjacent to nonmetropolitan areas. Similarly, we have shown that increased grade plays a significant negative role in outcomes in this more up-to-date SEER population. In contrast to the analysis of the older SEER data (2) there were some similarities and differences encountered with the larger patent dataset: Nama et al. found that tumor histology, age greater than 40 years, Black race, disease grade (undifferentiated), and the presence of distant metastases were all independently associated with increased mortality. In contrast, this study found that age under 65 years, race being White (versus non-White), treatment with either surgery, radiotherapy, or chemotherapy, and disease Grade (Grade I or II versus III or IV) all conveyed an increase in OS on univariate analysis, though on multivariable analysis only disease Grade and treatment with surgery maintained this trend. In summary, MMMT is a complex and aggressive sarcoma subtype that continues to be characterized by poor prognosis, however this updated analysis of the present work indicated that this malignancy possesses an at least partial and possibly synergistic responsiveness to radiotherapeutic, chemotherapeutic, and surgical modalities.

## Data availability statement

Publicly available datasets were analyzed in this study. This data can be found here: https://seer.cancer.gov/seerstat/download/; see methods section for query criteria.

## Ethics statement

Ethical approval was not required for the study involving humans in accordance with the local legislation and institutional requirements. Written informed consent to participate in this study was not required from the participants or the participants’ legal guardians/next of kin in accordance with the national legislation and the institutional requirements.

## Author contributions

NZ: Data curation, Writing – original draft, Writing – review & editing. AB: Data curation, Formal analysis, Investigation, Methodology, Project administration, Software, Supervision, Visualization, Writing – review & editing. VS: Conceptualization, Investigation, Methodology, Writing – original draft. MP: Writing – review & editing. GE: Writing – review & editing. ML: Project administration, Writing – review & editing, Investigation. MP: Project administration, Writing – review & editing, Methodology, Resources, Supervision, Validation.
